# Up-front systemic chemotherapy is a feasible option compared to primary tumor resection followed by chemotherapy for colorectal cancer with unresectable synchronous metastases

**DOI:** 10.1186/s12957-015-0570-1

**Published:** 2015-04-24

**Authors:** Hiroaki Niitsu, Takao Hinoi, Manabu Shimomura, Hiroyuki Egi, Minoru Hattori, Yasuyo Ishizaki, Tomohiro Adachi, Yasufumi Saito, Masashi Miguchi, Hiroyuki Sawada, Masatoshi Kochi, Shoichiro Mukai, Hideki Ohdan

**Affiliations:** Department of Gastroenterological and Transplant Surgery, Applied Life Sciences, Institute of Biomedical & Health Sciences, Hiroshima University, 1-2-3 Kasumi, Minamiku, Hiroshima 734-8551 Japan

**Keywords:** Colorectal cancer, Stage IV, Unresectable metastases, Surgery, Chemotherapy

## Abstract

**Background:**

In stage IV colorectal cancer (CRC) with unresectable metastases, whether or not resection of the primary tumor should be indicated remains controversial. We aim to determine the impact of primary tumor resection on the survival of stage IV CRC patients with unresectable metastases.

**Methods:**

We retrospectively investigated 103 CRC patients with stage IV colorectal cancer with metastases, treated at Hiroshima University Hospital between 2007 and 2013. Of these, those who had resectable primary tumor but unresectable metastases and received any chemotherapy were included in the study. We analyzed the overall survival (OS) and short-term outcomes between the patients who received up-front systemic chemotherapy (USC group) and those who received primary tumor resection followed by chemotherapy (PTR group).

**Results:**

Of the 57 included patients, 15 underwent USC and 42 PTR. The median survival times were 13.4 and 23.9 months in the USC and PTR groups, respectively (*P* = 0.093), but multivariate analysis for the overall survival showed no significant difference between the two groups (hazard ratio, 1.30; 95% confidence interval (CI), 0.60 to 2.73, *P* = 0.495). In the USC group, the disease control rate of primary tumor was observed in 12 patients (80.0%), but emergency laparotomy was required for 1 patient. Morbidity in the PTR group was observed in 18 cases (42.9%).

**Conclusions:**

The overall survival did not differ significantly between the USC and PTR groups. USC may help avoid unnecessary resection and consequently the high morbidity rate associated with primary tumor resection for stage IV CRC with unresectable metastases.

## Background

Medical treatment of colorectal cancer (CRC) has significantly improved over the past 10 years, mostly because of the introduction of combination chemotherapy protocols, and, more recently, new biological agents [1]. The median survival time of CRC patients has recently increased to over 2 years by using combinatorial therapy with molecular targeted agents such as bevacizumab, cetuximab, and panitumumab [2].

However, in stage IV CRC with unresectable metastases, whether or not resection of the primary tumor should be indicated remains controversial. Although it has been previously demonstrated that primary tumor resection or palliative surgery against symptomatic primary tumors is valuable, opinions vary on the need for resection of asymptomatic primary tumors [3,4]. The need for prophylactic resection of asymptomatic primary tumors to prevent emerging symptoms is debatable, and it is difficult to predict the contribution of such procedures to quality of life improvements, especially when the estimated survival time is limited [5]. Moreover, it should be considered that patients with advanced CRC might have a higher risk of surgical morbidity and mortality [6-9].

Recent advances in systemic chemotherapy may result in initially unresectable metastases becoming resectable, and consequently, resection of the primary tumor can be reassessed as part of a multidisciplinary therapeutic process, instead of palliative care alone [9-13]. Conversely, others have reported that, during up-front systemic chemotherapy, 57.1% to 97.1% patients did not require the additional surgery for morbidity including obstruction, perforation, bleedings, and so forth, [14-17] and that the mean interval between diagnosis and start of chemotherapy was 23.1 days [17]. As well as to avoid the unnecessary resection of primary tumor sometimes associated with surgical morbidity and mortality, up-front systemic chemotherapy was thought to be useful for earlier administration of chemotherapy for metastases that become life-threatening in the future and subsequently may contribute to prolong the survival outcomes.

Since the current literature does not offer confirmative evidence on the issue, we herein aimed to determine whether primary tumor resection followed by chemotherapy or up-front systemic chemotherapy provides a better prognosis in stage IV CRC with unresectable metastases.

## Methods

### Patients

**Figure 1:**
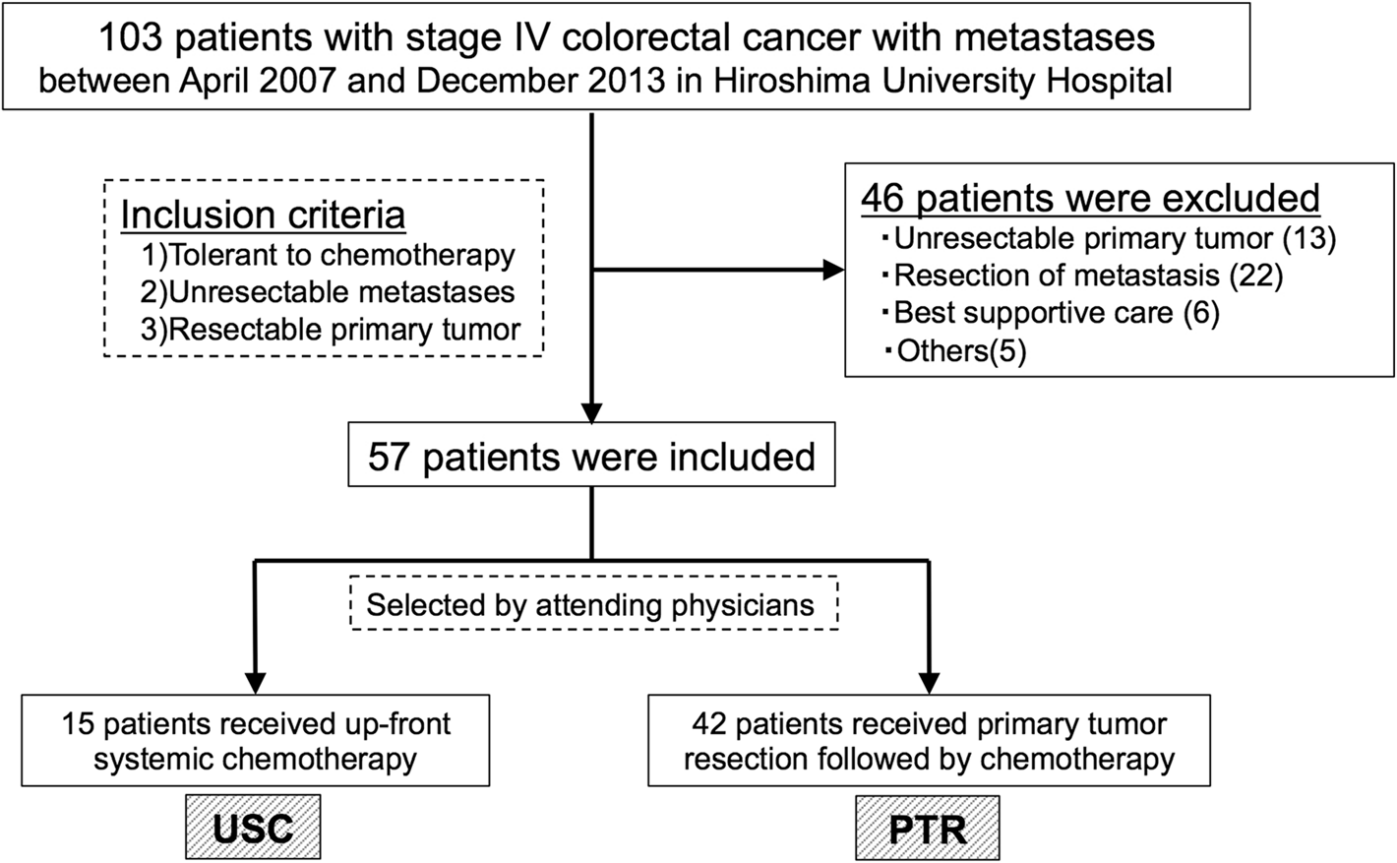
Inclusion criteria.

We retrospectively investigated 103 patients with stage IV CRC, who did not have symptomatic primary tumor requiring emergency surgery and thus contraindicating up-front systemic chemotherapy, treated at the Department of Gastroenterological and Transplant Surgery, Hiroshima University Hospital between April 2007 and December 2013. Of these, 13 patients with unresectable primary tumors due to multiple organ invasion and 22 patients with resectable or possibly resectable metastases were excluded from the study. Furthermore, 6 patients who were indicated for supportive care, and 5 patients with malignancies other than colorectal adenocarcinoma, including neuroendocrine carcinoma, appendiceal cystadenocarcinoma, squamous cell carcinoma of the anal canal, and multiple cancers under treatment, were also excluded, resulting in 57 patients with a resectable primary tumor but unresectable metastases being included in this study (Figure 1). Decision making of each treatment option was owing to the patients’ choice under sufficient informed consent from main surgeons and/or medical oncologists. Fifteen patients were treated with up-front systemic chemotherapy (USC) as initial therapy and 42 were treated with primary tumor resection (PTR) followed by chemotherapy.

This study was performed with permission of the Ethics Committee of the Hiroshima University.

### Chemotherapy

During the study period, almost all anti-cancer drugs indicated for CRC were available in Japan; 5-fluorouracil/leucovorin, capecitabine, S-1, oxaliplatin, irinotecan, bevacizumab, cetuximab (since August 2008), and panitumumab (since April 2010) were all administered to the patients, while regorafenib or trifluridine were not. As a general rule of our institute, oxaliplatin-based doublet chemotherapy (mFOLFOX6 or XELOX) with any molecular targeted agent was selected as first-line chemotherapy, and subsequently irinotecan-based doublet chemotherapy (FOLFIRI) with any molecular targeted agent was selected as second line, although chemotherapy regimens were finally decided on an individual basis by the attending medical oncologists. Bevacizumab was administered even when a primary tumor was present in the USC group, and administered at least 1 month after primary tumor resection in the PTR group, because of its possible role in delayed wound healing. Genotyping of Kirsten rat sarcoma viral oncogene (*KRAS*) exon 2 was performed for all cases from April 2010, when the Japanese Ministry of Health and Welfare approved its reimbursement for patients with health insurance. Before approval, the *KRAS* exon 2 was genotyped only when cetuximab or panitumumab was considered; therefore, the *KRAS* status was unknown in some cases. Cetuximab and panitumumab were offered only when a wild-type *KRAS* exon 2 genotype was confirmed. Other *RAS* genotypes were not studied.

### Statistical analyses

The following baseline characteristics were compared between the USC and PTR groups: age, sex, body mass index (BMI), Eastern Cooperative Oncology Group performance status (ECOG-PS) [18], American Society of Anesthesiologists score (ASA score), tumor location (colon or rectum), *KRAS* status, carcinoembryonic antigen (CEA), invasion depth and lymph node metastases classified according to the UICC-TNM stage (Union for International Cancer Control 7th edition [19]), sum of the longest diameters of the three largest metastases, number of metastases, number of organs with metastasis, and chemotherapy regimens (number of courses and anti-cancer drugs used). The operative procedures were also analyzed in both PTR and USC groups. Moreover, postoperative morbidity, response rate, disease control rate in both groups, and symptom occurrence rate after initiation of USC by primary tumor progression were studied. The results are reported as median and interquartile range for quantitative variables and as frequencies for categorical variables. Comparisons were conducted using Wilcoxon’s rank-sum tests for quantitative variables and Fisher’s exact tests or Pearson’s Chi-square tests for categorical variables.

Survival outcomes were analyzed as of September 2014 and were compared between the two groups using log-rank tests and summarized as Kaplan-Meier curves and hazards ratios (HRs) with 95% confidence intervals (CIs). Subsequently, multivariate analyses for survival were conducted using Cox proportional hazard models, including variables at *P* < 0.1 in the log-rank tests. The results of these univariate or multivariate analyses are presented as the odds ratio (OR) or HR and 95% CI with the corresponding *P* value.

All statistical analyses were performed using JMP 10 software (SAS Institute, Cary, NC, USA).

## Results

**Figure 2:**
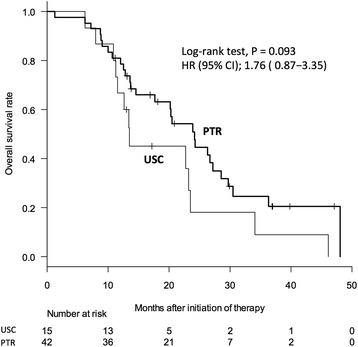
Overall survival between the USC and PTR groups. Median survival times were 13.4 and 23.9 months in USC and PTR, respectively, and overall survival was not significantly different in the two groups. USC: up-front systemic chemotherapy group. PTR: primary tumor resection group. HR: hazard ratio. CI: confidence interval.

On analyses of the baseline demographic and clinicopathological characteristics, sex and the primary tumor sites were found to significantly differ between the two groups, whereas other variables such as the age, BMI, ECOG-PS, ASA score, CEA, *KRAS* status, depth of invasion, lymph node metastasis, sum of the longest tumor diameter, number of metastases, number of organs with metastasis, peritoneal dissemination, and chemotherapy regimens did not significantly differ (Table 1). There were no differences with regard to the operative procedure offered to patients in both groups (Table 2). The median survival times were 13.4 months and 23.9 months for the USC and PTR groups, respectively, as determined by using the Kaplan-Meier analyses, and there was no statistical significance in overall survival (OS) (Figure 2). Moreover, the multivariate Cox regression analysis did not reveal any significant differences in survival between the two treatment options, whereas the *KRAS* status and number of organs with metastasis was found to be a significant independent prognostic factor for the survival of patients with CRC in the current study (Table 3).

**Table 1:** **Baseline demographic and clinicopathological characteristics**

**Characteristic**		**USC (** ***N*** **= 15)**	**PTR (** ***N*** **= 42)**	***P*** **value**
Follow-up duration (months)		13.4 (11.3 to 23.2)	19.2 (12.4 to 28.9)	0.232
Age (years)		63 (48 to 65)	61.5 (54 to 70.5)	0.568
Sex	Male	7	8	0.048
	Female	8	34	
BMI (kg/m^2^)		21.2 (16.9 to 25.5)	21.4 (19.8 to 23.1)	0.978
Performance status	0	14	39	1.00
	1	1	3	
	≥2	0	0	
ASA score	1	3	10	0.949
	2	11	29	
	3	1	3	
	4	0	0	
Primary tumor site	Right colon	1	13	0.005
	Left colon	3	18	
	Rectum	11	11	
KRAS status	Wild type	6	24	0.368
	Mutant or unknown	9	18	
CEA		23.1 (4.1 to 60.3)	41.5 (12.9 to 344)	0.074
Invasion depth	T2	0	1	0.337
	T3	6	17	
	T4a	9	20	
	T4b	0	4	
Lymph node metastasis	Negative	1	5	1.00
	Positive	14	37	
Sum of the longest diameters of metastases (mm)		61 (46 to 150)	75 (41.5 to 109)	0.697
Number of metastases		17 (6 to 52)	11.5 (5.5 to 28)	0.309
Number of organs with metastasis		2 (1 to 2)	2 (1 to 2)	0.899
Peritoneal dissemination		2 (13.3%)	7 (16.7%)	1.00
Total lines of chemotherapy		2 (2 to 3)	2 (1 to 3)	1.00
Use or any molecular target agents		13 (86.7%)	37 (88.1%)	1.00
Bevacizumab		12 (80.0%)	33 (78.6%)	1.00
Cetuximab and/or panitumumab*		6 (100%)	16 (66.7%)	0.155
Use of oxaliplatine combination		14 (93.3%)	36 (85.7%)	0.66
Use irinotecan combination		12 (80.0%)	26 (61.9%)	0.339
Conversion		1 (6.7%)	0 (0%)	0.263

USC: up-front systemic chemotherapy group. PTR: primary tumor resection group. BMI: body mass index. ASA score: American Society of Anesthesiologists score. KRAS: Kirsten rat sarcoma viral oncogene. CEA: carcinoembryonic antigen. Variables were statistically analyzed by Wilcoxon’s rank-sum test (quantitative variables), Fisher’s exact test (categorical, binary) or Chi-square test (categorical, more than three variables). *Use of cetuximab and/or panitumumab was presented as frequency in the KRAS exon 2 wild-type tumor only.

**Table 2:** **Surgical procedures offered to patients in the PTR and USC groups**

	**PTR (** ***N*** **= 42)**	**USC (** ***N*** **= 6)**	***P*** **value**
Surgical procedure, *n*			
Ileocecal resection	5	-	0.181
Right hemicolectomy	4	-	
Transverse colectomy	4	-	
Left hemicolectomy	2	-	
Sigmoidectomy	11	2	
Hartmann operation	3	1	
Abdominoperineal resection	5	-	
High anterior resection	3	-	
Low anterior resection	5	2	
Only ostomy creation	-	1	
Approach			0.591
Open	34	4	
Laparoscopic	8	2	
Open conversion, *n*	0	0	N.A.

USC: up-front systemic chemotherapy group. PTR: primary tumor resection group. N.A.: not available. Variables were statistically analyzed by Fisher’s exact test (categorical, binary) or Chi-square test (categorical, more than three variables).

**Table 3:** **Univariate and multivariate analysis on overall survival**

		**Univariate analysis**		**Multivariate analysis**	
**Factor**	***N***	**HR (95% CI)**	***P*** **value**	**HR (95% CI)**	***P*** **value**
Treatment					
PTR	42	Reference	0.093	Reference	0.495
USC	15	1.76 (0.87 to 3.35)		1.30 (0.60 to 2.73)	
Age					
<63 years	30	Reference	0.923	-	-
≥63 years	27	0.97 (0.52 to 1.80)			
Sex					
Male	15	Reference	0.116	-	-
Female	42	1.70 (0.84 to 3.24)			
BMI					
<22 kg/m^2^	33	Reference	0.627	-	-
≥22 kg/m^2^	24	0.85 (0.44 to 1.61)			
Tumor location					
Colon	35	Reference	0.280	-	-
Rectum	22	0.70 (0.35 to 1.32)			
KRAS status					
Wild type	30	Reference	0.001	Reference	0.004
Mutant or unknown	27	2.81 (1.49 to 5.31)		2.89 (1.41 to 6.02)	
CEA					
<5.1	10	Reference	0.822	-	-
≥5.1	47	1.09 (0.53 to 2.56)			
Invasion depth					
T1-3	24	Reference	0.0540	Reference	0.065
T4a, T4b	33	1.93 (0.99 to 3.87)		1.97 (0.96 to 4.24)	
Lymph node metastasis					
Negative	6	Reference	0.225	-	-
Positive	51	2.47 (0.89 to 10.2)			
Sum of the longest diameters of metastases					
<67 mm	29	Reference	0.184	-	-
≥67 mm	28	1.52 (0.82 to 2.87)			
Number of metastases					
<13	29	Reference	0.084	Reference	0.199
≥13	28	1.72 (0.93 to 3.24)		1.55 (0.79 to 3.09)	
Number of organs with metastasis					
1	22	Reference	0.010	Reference	0.037
≥2	35	2.52 (1.26 to 5.48)		2.14 (1.04 to 4.76)	
Peritoneal dissemination					
No	48	Reference	0.129	-	-
Yes	9	1.90 (0.76 to 4.16)			
Total lines of chemotherapy					
1 to 2	35	Reference	0.111	-	-
≥3	22	0.60 (0.31 to 1.13)			
Use of anti-VEGF antibody					
No	12	Reference	0.403	-	-
Yes	45	0.71 (0.34 to 1.69)			
Use of anti-EGFR antibody (KRAS wild patient only)					
No	8	Reference	0.197	-	-
Yes	22	2.22 (0.73 to 9.59)			

USC: up-front systemic chemotherapy group. PTR: primary tumor resection group. HR: hazard ratio. CI: confidence interval. BMI: body mass index. KRAS: Kirsten rat sarcoma viral oncogene. EGFR: epidermal growth factor receptor. CEA: carcinoembryonic antigen. VEGF: vascular endothelial growth factor.

The response rates to first-line chemotherapy were 33.3% and 42.9% in the USC and PTR groups, respectively (*P* = 0.558), while the disease control rates were 80.0% and 71.4%, respectively (*P* = 0.735). In the USC group, 3 patients (20.0%) experienced symptoms due to primary tumor after initiation of chemotherapy, resulting in additional surgery. For one of the three, emergency laparotomy was required because of perforative peritonitis caused by tumor necrosis after USC. On the other hand, 3 patients received primary tumor resection, not as palliation for symptoms caused by the primary tumors but as a multidisciplinary therapy after the primary tumors and metastases were controlled with up-front chemotherapy. In total, 6 patients received the surgery, and 3 patients (50%) experienced postoperative morbidity, including 2 surgical site infections (SSI), 1 anastomotic leakage, and 1 pneumonia.

In the PTR group, the morbidity was observed in 18 cases (42.9%), including 6 postoperative ileuses, 3 anastomotic leakages, 3 surgical site infections, and 10 cases with other complications such as liver function test abnormalities and delirium. Of these, 6 cases (14.3%) were categorized as > grade 3 complications.

## Discussion

In stage IV CRC with unresectable metastases, whether primary tumor resection followed by chemotherapy or up-front systemic chemotherapy provides a better prognosis remains controversial. Contrary to our result that the OS rates do not differ according to primary tumor resection in stage IV CRC with unresectable metastases, some previous reports have indicated a better survival benefit with primary tumor resection. In a recent meta-analysis of eight studies with available survival data, primary tumor resection was associated with longer OS, with an estimated median standardized difference of 6.0 months [11]. In another meta-analysis of 21 studies, primary tumor resection also contributed to longer OS; however, most of the studies included were retrospective (18 of 21) and chemotherapy regimens differed from each other. With regard to molecular targeted agents, only two study included bevacizumab and one cetuximab (with bevacizumab) [20]. Additionally, two recent sub-analyses of data from randomized trials [12,13] showed the survival benefits for patients who underwent primary tumor resection. Although these were discrepant to our result, it may be because stage IV disease included many complicated conditions, such as the resectability of primary tumor, the volume of metastatic tumor, the pace of progression, and so on. To minimize the influence of these complicated situations, only patients with stage IV disease with a resectable primary tumor but unresectable metastases were included in the current study. For the same purpose, we evaluated the sum of the longest diameters of the metastases, number of metastases, and number of organs with metastasis and confirmed that there were no statistical differences between the two groups.

Moreover, the rapid progress made in the therapeutic treatments for colorectal cancer should also be considered. For example, anti-epidermal growth factor receptor (*EGFR*) antibodies were not included or mentioned in most of these previous studies. While only one study has mentioned the use of cetuximab [13,20,21], which was added to combination therapy regimens comprising capecitabine, oxaliplatin, and bevacizumab, this regimen was not used as standard therapy, because no survival prolongation from adding cetuximab on bevacizumab has been demonstrated. It is notable that an anti-*EGFR* antibody was used in 73.3% of all patients who had *KRAS* wild-type tumors in the current study. Moreover, we did not observe any significant differences in the rates in which both cytotoxic and molecular targeted agents including anti-*EGFR* antibodies were used between the USC and PTR groups. Consequently, the influences by the varied chemotherapy regimens on the result might be minimal.

In addition to primary tumor resection, the *KRAS* mutation was found to have a negative prognostic value in our multivariate analysis, while a prognostic value of *KRAS* mutation on survival in metastatic CRC is controversial in the previous literatures [22]. *KRAS* mutation was not found to affect the survival in some studies [23-26]. However, most recently, worse OS rates were reported in patients with mutated *KRAS* compared to patients with wild-type *KRAS* in a sub-analysis of a previous randomized controlled study (RCT) with a large sample size [27]. The result of the current study is compatible with the most recent literature.

Because the OS rates did not significantly differ between the PTR and USC groups in the present study, it is important to evaluate the characteristics of these treatments to determine which leads to a better outcome. USC offers the possibility of avoiding potentially unnecessary primary tumor resection, and accordingly, the primary tumors were controlled in 80.0% of patients in the USC group in this study. However, it should be noted that emergency laparotomy in the USC group owing to perforation was performed in one case (6.7%). On the other hand, 42.9% of patients in the PTR group experienced morbidities, which is higher than the morbidity rates observed in patients undergoing elective surgery for stages 0 to III CRC at our hospital (28.7% in control group, Table 4). The high morbidity rate reported in the present study should raise some concerns. There were no statistical differences in each complication, such as postoperative ileus, anastomotic leakage, and SSI, but the number in every complication is larger in the PTR group. As the sum, morbidity is thought to be more frequent in the PTR group in the current study. In some previous reports [6-9], postoperative morbidity and mortality in PTR are reported to be higher than non-resection group such as ostomy and bypass. However, the reason for higher morbidity and mortality is still unclear. Further studies aiming to compare directly stage IV CRC to stages 0 to III should be required to estimate this issue in the future.

**Table 4:** **Complication in PTR compared with elective surgery for stages 0 to III in our hospital**

		**PTR (** ***N*** **= 42)**	**Control (** ***N*** **= 352)**	***P*** **value**
Mortality		0%	0%	N.A.
Morbidity	Overall	18 (42.9%)	98 (28.7%)	0.0498
	>Grade 3	6 (14.3%)	38 (10.7%)	0.445
	Postoperative ileus	6 (14.3%)	29 (8.2%)	0.244
	Leakage	3 (7.1%)	20 (5.7%)	0.731
	Surgical site infection	5 (11.9%)	32 (9.1%)	0.574

PTR: primary tumor resection group. Control: groups in which elective surgery for stages 0 to III CRC were performed between 2010 and 2012 in our hospital. N.A.: not available. Variables were statistically analyzed by Fisher’s exact test.

The limitation of the current study was its retrospective nature. The small number of patients, especially in the USC group, is also the limitation in our study. This is because the concept of USC is thought to emerge after the appearance of molecular targeted agents that potentially enable conversion therapies, and therefore, we have not experienced the large number of patients yet. Although our result may not be conclusive, it suggests that it is important to consider the benefits and disadvantages of both these treatments and to select an appropriate option on an individual basis until standard therapy might be determined in the future based on the results of ongoing RCTs.

## Conclusions

Our results here indicate that the OS did not differ significantly depending on the performance of primary tumor resection in stage IV CRC with unresectable metastases. Furthermore, up-front systemic chemotherapy may be valuable in avoiding unnecessary primary tumor resection and surgical morbidity.

## Abbreviations

ASA score: American Society of Anesthesiologists score
BMI: body mass index
CEA: carcinoembryonic antigen
CI: confidence interval
CRC: colorectal cancer
ECOG-PS: Eastern Cooperative Oncology Group performance status
EGFR: epidermal growth factor receptor
HR: hazard ratio
KRAS: Kirsten rat sarcoma viral oncogene
OR: odds ratio
OS: overall survival
PTR: primary tumor resection followed by chemotherapy
RCT: randomized controlled trial
SSI: surgical site infection
UICC: Union for International Cancer Control
USC: up-front systemic chemotherapy
